# Rationale and Design of the PREDICT-CCM Study: Predictive Value of Dobutamine Stress Echocardiography for Clinical Response to Cardiac Contractility Modulation Therapy in a Multicenter Italian Cohort

**DOI:** 10.3390/jcm15093223

**Published:** 2026-04-23

**Authors:** Francesco Zanon, Carlo Uran, Vincenzo Bonfantino, Natale Di Belardino, Antonio Lupo, Marzia Giaccardi, Procolo Marchese, Angelo Antonio Di Grazia, Luca Santini, Luigi Di Lorenzo, Giovanni Carreras, Luca Sgarra, Matteo Ziacchi, Leonardo Marinaccio, Luigi Mancini, Giovanni Bisignani, Mariateresa Manes, Stefano Guarracini, Amir Kol, Roberto Floris, Antonio Rossillo, Gabriele Zanotto, Lina Marcantoni, Franco Noventa

**Affiliations:** 1 Ospedale Santa Maria della Misericordia, 45100 Rovigo, Italy; lina.marcantoni@aulss5.veneto.it; 2 P. O. Anastasia Guerriero, 81025 Marcianise, Italy; carlura367@gmail.com; 3 Ospedale di Venere, 70131 Bari, Italy; enzobonfy@gmail.com; 4 Ospedale di Anzio, 00042 Anzio, Italy; natale.dibelardino@aslroma6.it; 5 Ospedale di Mirano, 30035 Mirano, Italy; antonio.lupo@aulss3.veneto.it; 6 P. O. Santa Maria Annunziata, 50012 Firenze, Italy; marzia.giaccardi@uslcentro.toscana.it; 7 Ospedale Gen. Prov. Mazzoni, 63100 Ascoli, Italy; procolo.marchese@gmail.com; 8 AOU Policlinico G.Rodolico-San Marco, 95123 Catania, Italy; angelodigrazia@alice.it; 9 P. O. G. B. Grassi, 00122 Ostia, Italy; lsantini@alice.it; 10 Ospedale San Rocco, 81037 Sessa Aurunca, Italy; luidilor@gmail.com; 11 Ospedale di Terni, 05100 Terni, Italy; giovannicarreras@libero.it; 12 Ospedale Miulli, 70021 Acquaviva Delle Fonti, Italy; sgarraluca@gmail.com; 13 Cardiology Unit, Cardiac Thoracic and Vascular Department, IRCCS Azienda Ospedaliero Universitaria di Bologna, 40138 Bologna, Italy; matteo.ziacchi@gmail.com; 14 Ospedale di Piove di Sacco, 35028 Piove di Sacco, Italy; leonardo.marinaccio@aulss6.veneto.it; 15 Ospedale San Paolo, 70123 Bari, Italy; drluigimancini@gmail.com; 16 Ospedale Ferrari, 87012 Castrovillari, Italy; giovanni.bisignani@virgilio.it; 17 Ospedale San Francesco, 87027 Paola, Italy; mteresa.manes@aspcs.it; 18 Casa di Cura Pierangeli e Spatocco, 65124 Pescara, Italy; stefanoguarracini@yahoo.it; 19 Ospedale San Camillo de Lellis, 02100 Rieti, Italy; a.kol@asl.rieti.it; 20 Ospedale Nostra Signora di Bonaria, 09037 San Gavino, Italy; roberto.floris@aslmediocampidano.it; 21 Ospedale S. Bortolo, 36100 Vicenza, Italy; antonio.rossillo@aulss8.veneto.it; 22 Ospedale Magalini, 37069 Villafranca di Verona, Italy; gabriele.zanotto@aulss9.veneto.it; 23 QUOVADIS Non-Profit Association, 35139 Padova, Italy

**Keywords:** heart failure, cardiac contractility modulation (CCM), dobutamine stress echocardiogram (DSE), contractile reserve

## Abstract

**Background/Objectives**: Heart failure (HF) is associated with substantial morbidity, impaired quality of life (QOL), and reduced functional capacity. In selected patients with symptomatic HF despite Optimal Medical Therapy (OMT), Cardiac Contractility Modulation (CCM) may be a therapeutic option. Identifying patients most likely to benefit from CCM remains an unmet need. The Predict-CCM study aims to evaluate long-term clinical and objective outcomes after CCM therapy and to assess the predictive value of pre-implant low-dose dobutamine stress echocardiography (LDDSE). **Methods and Results**: Predict-CCM is an independent, non-profit, multicenter, observational cohort study conducted in Italy, with both retrospective and prospective enrollment. The primary endpoint is the proportion of subjects with a clinical response to CCM at 12 months, defined as a ≥1-class reduction in NYHA class. Secondary clinical endpoints include reductions in HF-related hospitalizations, changes in QOL assessed by the Minnesota Living with Heart Failure Questionnaire (MLHFQ), and changes in NT-proBNP levels from baseline to follow-up. Outcomes will be evaluated in the overall cohort and in two subcohorts stratified by pre-implant LDDSE response: (1) reduction in left ventricular end systolic volume (LVESV) ≥ 15% (DeltaLVESV ≥ 15%); and (2) reduction in LVESV < 15% (DeltaLVESV < 15%). Assuming a 70% clinical response rate at 12 months, the estimated sample size is 120 patients. The study was approved by the Ethics Committee in March 2025. Enrollment will continue for 2 years, with a 12-month follow-up period after implant for each subject. **Conclusions**: This study may provide new criteria for patient selection and outcome assessment in CCM therapy. Left ventricular contractile reserve assessed by stress echocardiography may be a promising predictor of response.

## 1. Introduction

Heart failure (HF) remains associated with high mortality, impaired quality of life, and reduced functional capacity. It is among the most prevalent cardiovascular conditions worldwide and a major driver of healthcare utilization and costs. Despite advances in medical therapy, many patients continue to experience frequent hospitalizations and limitations in daily activities [1].

The prevalence of HF ranges from 1% to 3% among adults in industrialized countries and is expected to rise due to population aging and improved survival after diagnosis [2].

Contemporary international guidelines for HF with reduced ejection fraction (HFrEF) recommend early initiation of guideline-directed medical therapy, including ACE inhibitors or ARNIs, beta-blockers, mineralocorticoid receptor antagonists (MRAs), and SGLT2 inhibitors [3]. When medical therapy is insufficient or not tolerated, particularly in the presence of electrical conduction abnormalities (notably left bundle branch block), electrical therapies such as cardiac resynchronization therapy (CRT) may be indicated. CRT, when combined with pharmacologic therapy, improves symptoms, exercise capacity, and quality of life and reduces morbidity and mortality in appropriately selected patients.

### 1.1. Cardiac Contractility Modulation (CCM)

CRT is primarily indicated in patients with a wide QRS (>130 ms) and evidence of left bundle branch block [4]. However, the rate of non-response to CRT remains substantial (approximately 25–33% across studies) [5]. Randomized clinical trials have shown that Cardiac Contractility Modulation (CCM) is an option for patients with symptomatic HF despite optimized medical therapy who are ineligible for CRT [6]. CCM has also been evaluated in patients who do not respond to CRT [7].

CCM therapy is delivered by an implantable pulse generator connected to two right ventricular leads that deliver high-energy biphasic impulses during the myocardium’s absolute refractory period. The device does not provide pacing or anti-tachycardia therapies and can be used in patients who already have pacemakers or implantable cardioverter defibrillators.

The FIX-HF-4 [8], FIX-HF-5 [9,10], and FIX-HF-5C [11] trials demonstrated the safety and efficacy of CCM with respect to the following:

- Improvement in NYHA class;
- Improvement in quality of life, as measured by the MLHFQ;
- Improvement in functional capacity, as measured by the 6 min walk test (6MWT);
- Increase in peak oxygen consumption (VO2);
- Reduction in HF-related hospitalizations and, in some analyses, cardiovascular mortality.

CCM improves myocardial contractility by modulating intracellular calcium handling and produces both short- and long-term effects, including favorable changes in gene expression [12].

The Optimizer Smart system received CE Mark approval in October 2016 [13] and FDA approval in March 2019 for CCM therapy delivery.

In the 2021 ESC guidelines for acute and chronic heart failure, CCM is described as a therapy “under evaluation” for selected patients in NYHA class III/IV with a left ventricular ejection fraction (LVEF) of 25–45% and QRS < 130 ms [3]. In the 2024 Heart Failure Association (HFA) and the European Heart Rhythm Association (EHRA) clinical consensus statement, CCM is suggested as a therapy that multidisciplinary HF teams may consider for patients with persistent symptoms despite optimized therapy [14]. The iCARDIO Alliance Global Implementation Guidelines similarly include CCM among recommended interventions to improve symptoms, QOL, and exercise tolerance [15].

Economic evaluations suggest that, among selected patients, CCM therapy may be cost-effective compared with OMT alone over a lifetime horizon [16,17].

In carefully selected cases, CCM may also be considered for CRT non-responders when other options are limited [17]. A recent study reported that CCM improved outcomes in patients with HFrEF, NYHA class III, and moderately prolonged QRS duration (120–149 ms) [18].

In April 2025, the OPTIMIZER Smart Mini system received a European CE Mark for expanded indications in diastolic HF.

### 1.2. Pharmacological Stress Echocardiography

Pharmacological stress echocardiography is widely used to assess suspected ischemic heart disease and ischemic left ventricular dysfunction. The echocardiographic exam is performed during administration of pharmacologic agents that increase myocardial oxygen demand and contractility, thereby simulating exercise. The test may identify stress-induced ischemia.

Additional applications include quantifying contractile reserve in cardiomyopathies, assessing valvular and congenital heart disease, and evaluating diastolic function and pulmonary hypertension. Stress echocardiography is widely available, relatively inexpensive, and does not use ionizing radiation [19,20].

The most commonly used agents are dobutamine and dipyridamole. Dobutamine is a synthetic catecholamine that primarily stimulates β1-adrenergic receptors and, to a lesser extent, α1 and β2 receptors. Standard dobutamine stress echocardiography protocols are outlined in the American Society of Echocardiography (ASE) 2007 Guidelines [21]. Typically, the infusion begins at 5 μg/kg/min and is increased every 3–5 min to 10, 20, 30, and 40 μg/kg/min, aiming for 85% of the age-predicted maximal heart rate [22].

Low-dose dobutamine stress echocardiography (LDDSE) is used to assess myocardial viability and contractile reserve and is particularly informative for identifying a “biphasic response” [22]: a myocardial region increases contraction at a low inotropic dose but later becomes hypokinetic or akinetic at higher dobutamine doses. In LDDSE, dobutamine is administered in incremental doses (commonly 5, 10, and 20 μg/kg/min), each maintained for up to five minutes [20,22,23,24]. In patients with dilated cardiomyopathy, contractile reserve assessed by LDDSE is associated with functional recovery and long-term prognosis [25]. LDDSE has also been used to select candidates for CRT [26] and to predict reverse remodeling after CRT [27]. Many CRT studies have evaluated left ventricular reverse remodeling using low-dose dobutamine echocardiography. A positive response criterion is a reduction in left ventricular end-systolic volume (LVESV) of ≥ 15% [28,29,30].

## 2. Materials and Methods

### 2.1. Objective of the Study

The study aims to evaluate long-term clinical and objective outcomes in adult patients with symptomatic HF due to left ventricular systolic dysfunction who have received adequate medical therapy and undergone CCM treatment.

Endpoints will be assessed in the overall cohort and in two sub-cohorts stratified by pre-implant LDDSE response:

- “DeltaLVESV ≥ 15%” sub-cohort, defined by a decrease in LVESV of at least 15%.
- “DeltaLVESV < 15%” sub-cohort, defined by a decrease in LVESV of less than 15%.

#### 2.1.1. Primary Endpoint

- Proportion of subjects with a clinical response to CCM at 12 months (≥1 NYHA class reduction)

#### 2.1.2. Secondary Clinical Endpoints

1. Reduction in the number of hospitalizations, Emergency Department visits, or day-hospital admissions lasting more than 4 h (e.g., those requiring intravenous inotropes) compared with the previous year;
2. Change in the quality-of-life score, estimated using the “Quality of Life Questionnaire with Heart Failure—Minnesota” (MLHFQ), from baseline to the end of follow-up [31];
3. Change in walk distance between baseline and the end of follow-up in the walk test (6MWT) (optional);
4. Change in NT-proBNP level from baseline to the end of follow-up.

#### 2.1.3. Secondary Echocardiographic Endpoints

1. The proportion of subjects with a LVESV reduction of ≥15% on echocardiography at the end of follow-up compared with preimplantation;
2. The proportion of subjects with a ≥20% increase in velocity time integral (VTI) between preimplantation and the end-of-follow-up echocardiography;
3. The proportion of subjects with a ≥20% increase in ejection fraction (LVEF) between preimplantation and the end-of-follow-up echocardiography;
4. The proportion of subjects with progression or improvement in mitral regurgitation (MR), classified as mild, moderate, or severe.

#### 2.1.4. Secondary Safety Endpoints

1. The proportion of subjects who, during follow-up, will undergo cardiac resynchronization therapy (CRT) implantation;
2. The proportion of subjects who, during follow-up, will undergo left ventricular assist device (LVAD) implantation;
3. The proportion of subjects who will receive a cardiac transplant during follow-up;
4. The proportion of subjects who will die from HF during follow-up (also compared with the predicted mortality by the MAGGIC score [32]);
5. Assessment of arrhythmic burden (for a patient with an implantable cardioverter-defibrillator (ICD): number of ventricular tachycardia episodes treated with ATP/shock; for a patient with a pacemaker/ICD/loop recorder: percentage of time spent in atrial fibrillation);
6. Rate of procedure-related adverse events;
7. Procedure and fluoroscopy times;
8. Rate of reoperations (lead revision/replacement/infection).

### 2.2. Study Population

#### 2.2.1. Inclusion Criteria

- Subjects of both sexes aged ≥ 18 years;
- Ability to understand and sign informed consent, including consent to process sensitive personal data;
- Symptomatic HF despite optimal medical therapy (OMT);
- Reduced left ventricular systolic function (EF < 50%);
- Candidates for CCM implantation according to the European Society of Cardiology 2021 Guidelines on heart failure and the CE mark approval provision [3];
- At least one HF-related hospitalization, Emergency Department visit, or day-hospital admission lasting >4 h (e.g., intravenous infusion of inotropes) in the year before implantation.

#### 2.2.2. Exclusion Criteria

- Life expectancy < 1 year due to non-cardiac comorbidities;
- Contraindications to CCM implantation (e.g., absence of suitable vascular access, active infection, severe coagulopathies, mechanical tricuspid valve);
- Contraindications to pharmacologic stress echocardiography (e.g., decompensated HF, acute myocardial infarction, acute myocarditis/pericarditis, critical aortic stenosis, severe left ventricular outflow obstruction, aortic dissection, uncontrolled severe arrhythmias, known hypersensitivity to dobutamine, intraventricular thrombi) [23].

### 2.3. Study Design

PREDICT-CCM is a multicenter, independent, nonprofit, retrospective, and prospective observational cohort study. The promoter is the recognized nonprofit association QUOVADIS (Padua, Italy). Dr. Francesco Zanon (S. Maria della Misericordia Hospital, ULSS5 Polesana, Rovigo, Italy) coordinates the study. Participating centers are experienced in advanced HF care and CCM implantation.

Patients receiving a CCM implant from 1 July 2024 to the start of the study will be included in the retrospective cohort; those implanted thereafter will be enrolled prospectively.

Enrollment will continue for 2 years, and each subject will be followed for 12 months after implantation.

Clinical and objective evaluations and data collection in the eCRF will occur at enrollment, at implantation, at least once between months 1 and 6, and 12 months after CCM implantation.

The strategic LDDSE procedure was thoroughly discussed with the co-investigators to ensure uniformity and standardization across centers. A written protocol was provided to standardize procedures. Dobutamine is administered via incremental infusions of 5, 10, and 20 g/kg/min, with a maximum of 5 min per dose. The procedure is considered complete if the heart rate increases by 10%. Blood pressure and ECG monitoring are required at rest and at the end of each phase of the protocol. The infusion is interrupted if any of the following events occur: arrhythmia, angina, hemodynamic decompensation, ECG abnormalities, or left ventricular wall motion abnormalities in at least two segments. During dobutamine stress echocardiography, reverse remodeling of the left ventricle is assessed using two-dimensional echocardiography.

**Figure 1:**
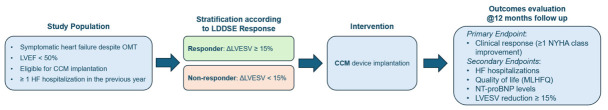
Study design and patient flow. Patients with symptomatic heart failure despite optimal medical therapy (OMT) undergo a baseline evaluation, including low-dose dobutamine stress echocardiography (LDDSE). Based on the change in LV end-systolic volume (LVESV), patients are stratified into responders (ΔLVESV ≥ 15%) and non-responders (ΔLVESV < 15%). All patients then undergo implantation of a Cardiac Contractility Modulation (CCM) device and are followed for 12 months. The primary endpoint is clinical response, defined as an improvement of at least one NYHA class. Secondary endpoints include heart failure hospitalizations, quality of life, NT-proBNP levels, and LVESV reduction.

The study design and patient flow are schematically depicted in Figure 1.

### 2.4. Statistical Analysis

#### 2.4.1. General Statistical Methods

All variables will be analyzed descriptively using appropriate statistical methods: categorical variables will be summarized with frequency tables, and continuous variables with sample statistics (e.g., mean, median, standard deviation, minimum and maximum values, and 25th and 75th percentiles).

The selected baseline covariates may be compared between the two subcohorts using appropriate statistical tests for discrete and continuous variables.

Unless otherwise specified, all statistical tests will be 2-tailed at a 5% significance level.

All primary and secondary analyses will be conducted in the modified intention-to-treat population (ITT), comprising all enrolled subjects who have undergone CCM implantation.

Subjects who are enrolled but withdraw consent before undergoing the CCM implant procedure may be replaced, even if they retain their unique identification code.

Subjects who leave the observation without reaching the endpoint will be considered censored and at risk during the observation period.

For those not present for clinical evaluations at the control visits between the 1st and 6th months and at the 12th month of observation, every effort will be made to determine a possible outcome of interest for the study and to make them assessable as endpoints.

The overall sample size is justified by the hypothesis parameters and determined by the primary endpoint to ensure adequate statistical power.

The primary endpoint is the proportion of subjects with clinical response to CCM therapy at 12 months (NYHA class reduction ≥ 1 class).

#### 2.4.2. Hypotheses

Based on an analysis of the on-topic scientific literature and our field experience, we hypothesize that:

1. Across all subjects, the proportion with clinical response to CCM therapy at 12 months of follow-up (NYHA class reduction ≥ 1 class) will be approximately 70%.
2. The proportion of subjects with a positive response to LDDSE before CCM implantation (i.e., a decrease in LVESV of at least 15%) will be 80%.

#### 2.4.3. Sample Size Estimation

When the proportion of subjects with a clinical response to CCM therapy at 12 months is 70%, a sample size of 120 will yield an acceptable two-sided 95% confidence interval with a width of 17%.

Under Hypothesis 2, the two sub-cohorts will consist of: 80 subjects in the “DeltaLVESV ≥ 15%” group, with an assumed clinical response to CCM therapy at 12 months of about 80%, and 40 subjects in the “DeltaLVESV < 15%” group, with an assumed clinical response to CCM therapy at 12 months of about 40%. We should have adequate power to evaluate the prognostic contribution of a positive LDDSE test, even in the presence of minor variations in observed responses relative to the expected ones (see protocol).

The power was estimated by PASS v.11 (NCSS Inc., Kaysville, UT, USA).

#### 2.4.4. Statistical Analysis Specifications

The study is primarily descriptive. Because the inclusion of both retrospective and prospective cohorts may introduce selection and confounding biases, these will be considered, and appropriate adjustments (propensity score and multivariate analyses) will be made to mitigate them. All possible efforts will be made to minimize missing data. If some variables have a high number of missing values (more than 20%), we will consider using multiple imputation (MICE). All these strategies will be detailed in the Statistical Analysis Plan (SAP) before the study ends.

#### 2.4.5. Primary Endpoint Data Analysis

The proportion of subjects with a clinical response to CCM therapy at 12 months (NYHA reduction ≥ 1 class) will be estimated, with 95% CIs, for the entire cohort and the two subcohorts. The crude Odds Ratio for clinical response between the subcohorts (“DeltaLVESV ≥ 15%” versus “DeltaLVESV < 15%”) will be estimated with its 95% CIs, and Fisher’s exact test will assess the association between the two factors.

A univariate logistic regression will assess associations between risk factors and primary efficacy endpoints and provide appropriate Odds Ratio estimates for other clinical or objective descriptors characterizing the enrolled subjects. Descriptors significantly associated (*p* < 0.10) with clinical response in the univariate analysis will be included in the multivariable regression and further selected using the standardized Forward Stepwise Wald method (with in- and out-thresholds at *p* = 0.10).

#### 2.4.6. Secondary Endpoints Data Analysis

All continuous secondary endpoints will be summarized using the number of non-missing observations, mean ± standard deviation, median, and range (minimum-maximum). All categorical secondary endpoints will be tabulated with counts and proportions, along with 95% CIs. A paired *t*-test will be used for within-group comparisons, and an equivalent non-parametric test, such as the Wilcoxon signed-rank or sign test, will be used if the paired *t*-test assumptions are violated. Between-group comparisons will be performed using a *t*-test or an equivalent non-parametric test, such as the Wilcoxon rank-sum or Kolmogorov–Smirnov test, when the *t*-test assumptions are violated.

All categorical secondary endpoints will be tabulated by occurrence and percentage. Fisher’s exact test will then be used to compare the groups. Measures to mitigate and correct for multiple tests, such as the Holm–Bonferroni method, will be applied. This decision will be documented in the SAP.

All statistical analyses will be performed using SPSS Statistics, version 26 or later (IBM Corp., Armonk, NY, USA), and STATISTICA v12 (StatSoft, Tulsa, OK, USA).

### 2.5. Current Status

The Territorial Ethics Committee “Southwest Veneto Area” (CET-ASOV) has approved the study for 45 Italian centers.

As of March 2026, 70 patients had been enrolled. Participating centers use an ‘Electronic Data Capture’ platform based on the ‘Research Electronic Data Capture’ (REDCap, [33] by Vanderbilt University and the ‘REDCap Consortium’) for data collection and data quality monitoring.

The PREDICT-CCM study is registered on the ClinicalTrials.gov website: NTC06973902.

## 3. Discussion

The Predict-CCM study aims to expand the understanding of the role of CCM in treating HF with left ventricular systolic dysfunction in patients who remain symptomatic despite OMT. Interest in CCM has grown in recent years because it improves myocardial contractility through non-excitatory electrical impulses, offering an additional option for a population with limited alternatives [34].

The ongoing AIM-HIGHer randomized controlled trial will provide additional insights into the efficacy and safety of CCM in patients with mildly reduced or preserved ejection fraction. Furthermore, next-generation devices that integrate CCM technology with ICD functionality may offer more comprehensive management for patients at risk of arrhythmic events [35].

Although previous studies have demonstrated that CCM improves functional capacity, quality of life, and selected clinical outcomes [8,9,10,11], identifying the patients most likely to benefit remains a major challenge. In a single-center pilot study, the echocardiographic response to Levosimendan infusion appeared to predict echocardiographic and clinical response to CCM [36].

The present study addresses the need to identify patients by evaluating whether pre-implant assessment of left ventricular contractile reserve using LDDSE can predict response to CCM. The underlying hypothesis is that the left ventricular response to pharmacologic inotropic stimulation reflects residual contractile capacity and may therefore correlate with subsequent clinical benefit from CCM. Stratifying patients into two subgroups—DeltaLVESV ≥ 15% and DeltaLVESV < 15%—will allow evaluation of whether left ventricular end-systolic volume reduction during stress testing predicts clinical and echocardiographic outcomes at follow-up.

The selected endpoints—improvement in NYHA class, reduction in HF-related hospitalizations, changes in quality-of-life scores (MLHFQ), and changes in NT-proBNP—capture complementary dimensions of the HF burden and treatment impact. The primary endpoint, focused on NYHA class, emphasizes a clinically meaningful improvement from the patient’s perspective, while the secondary outcomes will provide more detailed insights into the therapy’s overall impact. Moreover, the multicenter design and the mixed retrospective–prospective approach enhance generalizability to real-world practice.

Nevertheless, some inherent limitations should be acknowledged. The absence of a randomized control group may limit the ability to attribute observed effects solely to CCM therapy. Additionally, inter-operator variability in echocardiographic assessment, although minimized by standardized protocols, may affect subgroup classification based on contractile reserve. Despite these considerations, the projected sample size of 120 patients and the 12-month follow-up period are expected to yield robust data for evaluating the associations of interest.

If the study hypotheses are confirmed, Predict-CCM may offer new guidance for refining patient selection for CCM therapy and for using contractile reserve as a practical predictive and prognostic marker. Using a widely available tool such as stress echocardiography could also improve the reproducibility of selection criteria across centers, ultimately improving the quality of care.

In conclusion, the Predict-CCM study has the potential to address a significant gap in the management of advanced heart failure and to provide important insights to optimize CCM use and maximize its clinical benefit. These results may mark a meaningful step toward precision medicine in electrical therapies for heart failure.

## 4. Study Limitations

Combining retrospective and prospective studies introduces the risk of selection bias and confounding. However, given the study’s observational nature, this approach was adopted to better reflect real-world clinical practice. During the statistical analysis, the potential risk arising from the two populations was taken into account, and appropriate adjustments (propensity score and multivariate analyses) were made to mitigate potential bias.

To minimize inter-operator variability across centers, a standardized protocol for the LDDSE procedure was shared with Principal Investigators. Echocardiographic test results could not be evaluated by a central core lab because of heterogeneity in the echocardiographic equipment used.

## 5. Conclusions

The Predict-CCM study aims to provide new insights into the clinical effectiveness of CCM therapy for patients with symptomatic HF and left ventricular systolic dysfunction who remain inadequately controlled despite OMT. By integrating pre-implant assessment of contractile reserve using LDDSE, the study aims to identify reliable predictors of response to CCM therapy.

A comprehensive assessment of functional status, quality of life, biomarkers, and hospitalizations will clarify the multidimensional impact of CCM in a real-world population. The planned subgroup analysis based on changes in left ventricular end-systolic volume during stress testing may inform the development of more precise, individualized criteria for patient selection.

If the study hypotheses are confirmed, the findings could support incorporating contractile reserve assessment into routine pre-implantation evaluation, improving therapeutic decision-making and maximizing the clinical benefit of CCM therapy. Ultimately, Predict-CCM may help advance a more personalized approach to the electrical treatment of HF, guiding clinicians toward more targeted, evidence-based use of this emerging therapeutic modality.

## Data Availability

No new data were created or analyzed in this study.

## Abbreviations

The following abbreviations are used in this manuscript:

HF	Heart Failure
QOL	Quality Of Life
OMT	Optimal Medical Therapy
CCM	Cardiac Contractility Modulation
NYHA	New York Heart Association
LDDSE	Low-Dose Dobutamine Stress Echocardiography
MLHFQ	Minnesota Living with Heart Failure Questionnaire
LVESV	Left Ventricular End Systolic Volume
HFrEF	HF with reduced Ejection Fraction
MRAs	Mineralocorticoid Receptor Antagonists
CRT	Cardiac Resynchronization Therapy
6MWT	6-Minute Walk Test
VTI	Velocity Time Integral
LVEF	Left Ventricular Ejection Fraction
MR	Mitral Regurgitation
LVAD	Left Ventricular Assist Device
ICD	Implantable Cardioverter-Defibrillator
ITT	Intention-To-Treat
